# Limonin, an AMPK Activator, Inhibits Hepatic Lipid Accumulation in High Fat Diet Fed Mice

**DOI:** 10.3389/fphar.2022.833705

**Published:** 2022-01-24

**Authors:** Si-wei Wang, Tian Lan, Hang-fei Chen, Hao Sheng, Chun-yi Xu, Li-feng Xu, Fang Zheng, Feng Zhang

**Affiliations:** ^1^ Core Facility, The Quzhou Affiliated Hospital of Wenzhou Medical University, Quzhou People’s Hospital, Quzhou, China; ^2^ Zhejiang Chinese Medical University, Hangzhou, China; ^3^ Zhejiang University School of Medicine, Hangzhou, China

**Keywords:** limonin, AMPK, lipid accumulation, NAFLD, SREBP

## Abstract

NAFLD is the most prevalent liver disease in human history. The treatment is still limited yet. In the current study, we reported that limonin inhibited hepatic lipid accumulation and fatty acid synthesis in HFD fed mice. Using AMPK inhibitor and AMPK deficient *C. elegans*, we revealed the effect was dependent on the activation of AMPK. We found that limonin activated AMPK through inhibition of cellular energy metabolism and increasing ADP:ATP ratio. Furthermore, the treatment of limonin induced AMPK mediated suppression of the transcriptional activity of SREBP1/2. Our study suggests that limonin may a promising therapeutic agent for the treatment of NAFLD.

## Introduction

Non-alcoholic fatty liver disease (NAFLD), which is also called metabolic associated fatty liver disease (MAFLD) (Eslam et al., 2020), is characterized by pathological accumulation of triglycerides (TG) and other lipids in hepatocytes (Heeren and Scheja 2021; Ooi et al., 2021). It can progress to nonalcoholic steatohepatitis (NASH) and fibrosis, which eventually lead to liver cirrhosis, hepatocellular carcinoma. Although a variety of small molecule chemical drugs are undergoing clinical trials (Zhu et al., 2021), the treatment for NAFLD is still limited.

AMP-activated protein kinase (AMPK) is a key metabolic regulator that senses energy status and controls energy expenditure and storage, whose activation has been proposed to be therapeutically beneficial for the treatment of NAFLD (Garcia et al., 2019; Zhao et al., 2020). AMPK is activated in response to energy stress by sensing increases in AMP: ATP and ADP: ATP ratios and inhibited by adenosine triphosphate (ATP) (Garcia and Shaw 2017). Liver specific AMPK knockout can aggravate hepatic lipid accumulation, steatosis, inflammation, fibrosis and hepatocyte apoptosis (Garcia et al., 2019; Zhao et al., 2020). Hepatic activation of AMPK by the synthetic polyphenol protects against hepatic steatosis by suppressing sterol regulatory element binding protein 1 (SREBP1) activity (Li et al., 2011). Activating AMPK also inhibit hepatic cholesterol synthesis by suppression of SREBP2 activity (Tang et al., 2016). Our previous study showed that activation of AMPK by flavonoids could ameliorate hepatic steatosis in mice (Wang et al., 2020a).

**FIGURE 1:**
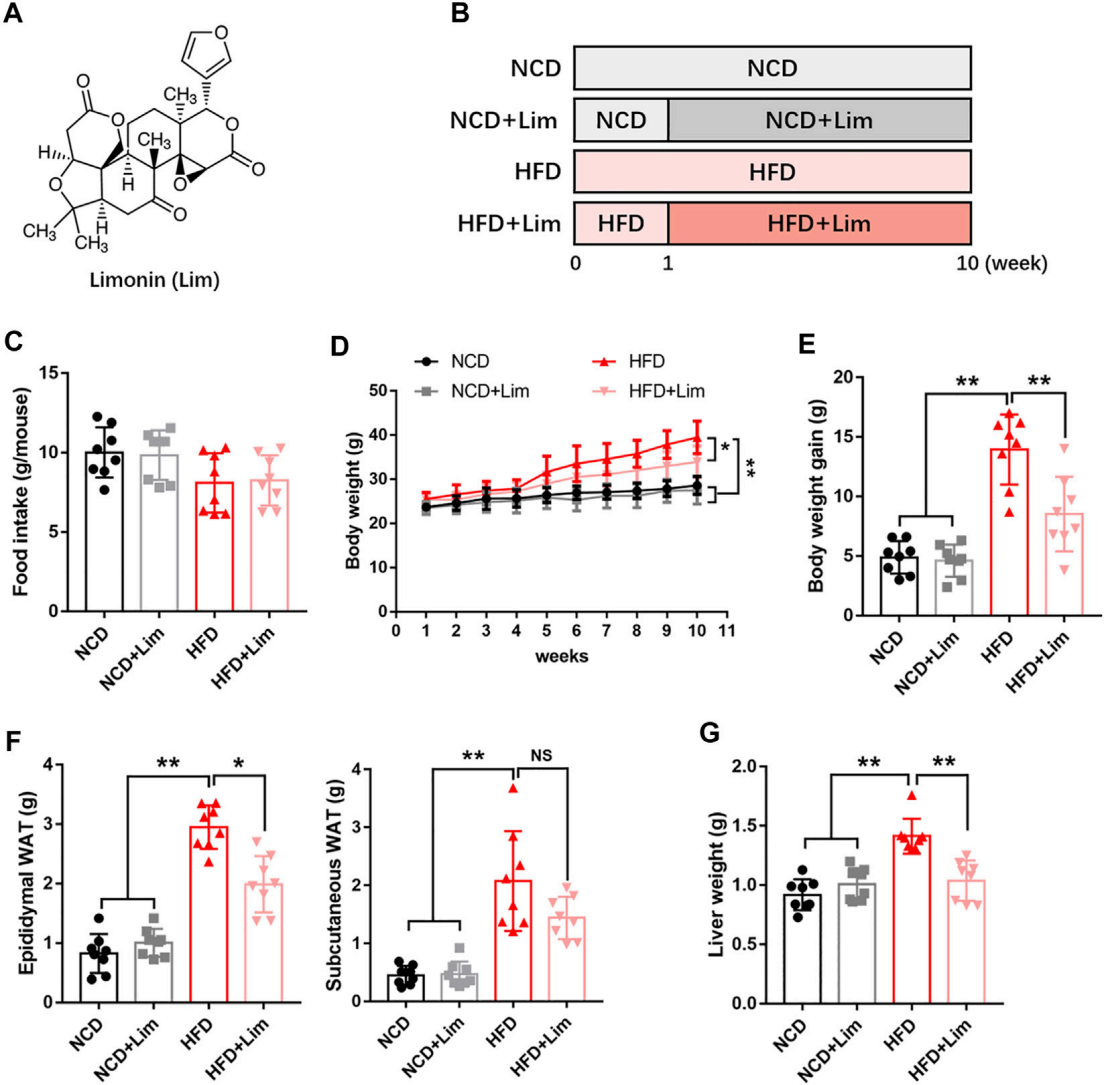
Lim inhibits the increase of body weight, adipose weight and liver weight induced by HFD in mice. **(A)** The chemical structure of Lim. Its molecular weight is 470.53. **(B)** The schematic diagram for HFD-induced fatty liver and Lim administration. C57BL/6 mice were fed either a chow diet as NCD or HFD for 10 weeks to induce fatty liver. Mice were treated with daily oral doses of Lim (50 mg/kg) from the second week of HFD diet feeding. Water was gavaged as control. **(C)** Food intake was calculated the average food intake of each mouse from the 2nd week to the 10th week. **(D)** Body weight curve. **(E)** Body weight gain. **(F)** Weight of epididymal white adipose tissue and subcutaneous white adipose tissue. **(G)** Weight of liver. Data were expressed as the mean ± SD (*n* = 8). ^*^ *p* < 0.05, ^**^ *p* < 0.01; NS, no significance.

Limonin (Figure 1A), a tetracyclic triterpenoid compound, is a secondary metabolite with high biological activity in plants (Fan et al., 2019). It is abundant in many traditional Chinese medicines and fruits (Fan et al., 2019). It has been recognized as one of the most beneficial and active components of medicinal foods (Gu et al., 2019). In recent years, pharmacological investigations have uncovered various bioactivities of limonin including anti-cancer (Chidambara Murthy et al., 2021), anti-inflammatory (Yang et al., 2021), anti-oxidation (Yu et al., 2005) and liver protection activity (Yang et al., 2020). Limonin reduces LDL cholesterol in HepG2 cells (Bhathena and Velasquez 2002) and regulates the expression of genes related to lipid metabolism in mice (Fan et al., 2019). However, the effect of limonin in hepatic lipid metabolism and the mechanism is still unclear.

Here, we reported that limonin inhibited hepatic lipid accumulation and fatty acid synthesis in HFD-induced mice. Further mechanistic studies revealed that limonin suppressed the transcriptional activity of SREBP1/2 by activating AMPK. In general, our study reveals that limonin may a promising therapeutic agent for the treatment of NAFLD.

## Materials and Methods

### Materials

Limonin (Lim, CAS# 1,180-71-8, HPLC≥95%) was purchased from TCI (Shanghai) Development Co., Ltd., China. Compound C (#B3252) was purchased from ApexBio, United States. Antibody sources are as follows: phospho-AMPKα (#2535, Cell Signaling Technology), AMPKα (#5831, Cell Signaling Technology), phospho-ACC (#3661, Cell Signaling Technology), ACC (#3662, Cell Signaling Technology), phospho-LKB1 (#3055, Cell Signaling Technology), LKB1 (#3050, Cell Signaling Technology), phospho-CAMKK2 (#12818, Cell Signaling Technology), CAMKK2 (#16810, Cell Signaling Technology), PP2A (#2038, Cell Signaling Technology), PP2C (#3549, Cell Signaling Technology), phospho-TAK1 (#4508, Cell Signaling Technology), TAK1 (#5206, Cell Signaling Technology), SREBP1 (ab28481, Abcam), SERBP2 (ab30682, Abcam), β-actin (#3700, Cell Signaling Technology). Commercial kits used in measurement of plasma parameters are as follows: triglyceride (TG), total cholesterol (TC) assay kits were purchased from Dongou Diagnostics Co., Ltd., Zhejiang, China; alanine aminotransferase (ALT), aspartate aminotransferase (AST), alkaline phosphatase (ALP), non-esterified fatty acid (NEFA), high-density lipoprotein cholesterol (HDL-C), and low-density lipoprotein cholesterol (LDL-C) assay kits were purchased from Nanjing Jiancheng Bioengineering Institute, China.

### Animal Experiments

All animal experiments were approved by the Animal Care and Use Committee of Zhejiang Chinese Medical University, where the experiments were conducted. 32 C57BL/6 male mice (eight-week-old), which were purchased from GemPharmatech Co., Ltd., Jiangsu, China (license number of animal production: SYXK 2015-0001), were housed two per cage in a temperature and humidity-controlled room with a 12:12 h light/dark cycle. After 1 week of acclimation, the mice were randomly separated into four groups: 1) NCD group: mice (*n* = 8) were orally administered with ultrapure water as control vehicle and fed with 10% Kcal high-fat, 7% sucrose control diet match D12492 (Research diet D12450J, Research Diet, NJ); 2) NCD + Lim group: mice (*n* = 8) were orally administered with limonin (Lim, 50 mg/kg/day) and fed with 10% Kcal high-fat, 7% sucrose control diet match D12492 (Research diet D12450J, Research Diet, NJ); 3) HFD group: mice (*n* = 8) were orally administered with ultrapure water as control vehicle and fed with 60% Kcal high-fat diet (Research diet D12492, Research Diet, NJ); 4) HFD + Lim group: mice (*n* = 8) were orally administered with limonin (Lim, 50 mg/kg/day) and fed with 60% Kcal high-fat diet (Research diet D12492, Research Diet, NJ). As described, the Lim administration started from the second week and the experiment lasted for 10 weeks in total (Figure 1B). Body weight and food intake were recorded weekly. At the end of the study, the blood samples were collected and the plasma parameters were detected using the indicated kits according to the manufacturers’ instructions. The tissues were dissected, weighed, immediately frozen in liquid nitrogen and stored at −80°C.

### Oral glucose tolerance tests and insulin tolerance test

OGTT and ITT were conducted at 9 weeks of the experiment. After fasting for 6 h, mice were oral d-glucose (2 g/kg) or i. p. injected with insulin (0.75 U/kg). Blood glucose levels were measured at 0, 30, 60, 90, and 120 min, which was measured by tail vein using a standard glucometer (Johnson & Johnson, United States).

### Histology Examination

Mouse liver tissues were fixed in fixed in 4% v/v phosphate-buffered formaldehyde, embedded in paraffin, sectioned, and stained with hematoxylin and eosin (H&E) according to our described previously (Bai et al., 2019; Wang et al. 2020a; Wang et al. 2021a). Lipid droplets were visualized by Oil Red O (Solarbio Life Science, China) staining. The NAFLD activity score (NAS) and the intensity of Oil Red O analysis were also based on the method according to our previous researches (Wang et al., 2020a).

### Cell Culture and Treatment

The mouse normal hepatic cell line AML12 (obtained from the Shanghai Bank of Cell Lines) was routinely cultured in Dulbecco’s Modified Eagle’s Medium (DMEM/F-12) containing 10% fetal bovine serum (FBS). Human hepatoma HepG2 cell line (obtained from the Shanghai Bank of Cell Lines) was cultured in Dulbecco’s Modified Eagle’s Medium (DMEM) containing 10% fetal bovine serum (FBS). To establish a hepatic lipid accumulation model using AML12 cells, we used 0.4 mM palmitic acid (PA) after starving in serum-free DMEM for 24 h.

### Immunofluorescence Staining

To visualize the expression and localization of SREBP1 and SREBP2 in cells, AML12 cells were incubated with anti-SREBP1 (1:100) or anti-SREBP2 (1:100) antibody at 4°C overnight. Then the cells were stained by Alexa Fluor 488 secondary antibody for 1 h at room temperature. The fluorescence was visualized by a SUNNY RX50 fluorescence microscope. An average score of the immunofluorescence was calculated as described previously (Wang et al. 2018; Wang et al. 2019).

### *C. elegans* Strain and Treatment

Wild-type N2 *C. elegans* and aak-2 (ok524) X mutant strains were obtained from the Caenorhabditis Genetics Center. All worms were fed on *Escherichia coli* OP50 lawn and raised at 20°C on nematode growth medium (NGM) agar plates. The yielding eggs were hatched in M9 buffer overnight at 20°C to obtain the age-synchronized L1 worms. The synchronized populations were further incubated on NGM plates which were pretreated with 100 μM Lim (dissolved in M9 buffer) for 6 days.

### *In vitro* Lipid Accumulation

Lipid accumulation in hepatocytes or *C. elegans* was visualized by Oil Red O staining or quantified by commercial kits (TC and TG), according to the manufacturer’s instructions.

### ADP/ATP Ratio Measurement

Cells were treated with different concentrations of limonin dissolved in DMSO for 2 h. ADP/ATP ratio was measured by ADP/ATP Ratio Assay Kit (#ab65313, Abcam, Burlingame, CA) according to the manufacturer’s instruction.

### Plasmids and Transfection

AMPK-DN was cloned from pMIGR-AMPK-KD (Addgene, Cat #27296) into pcDNA-3xFlag plasmid. The fragment of AMPKγ2 was cloned from pGEM-PRKAG2 (#HG16130-G, SinoBiological, Beijing, China) and constructed into pLKO-puro FLAG plasmid. Mutagenesis was performed using the Hieff Mut™ Site-Directed Mutagenesis Kit (YEASEN, Shanghai, China) according to the manufacturer’s instruction. The transfection was performed using Lipofectamine 3000 reagent from Life Technologies (Carlsbad, CA) according to our described previously (Wang et al., 2021b).

### Quantitative Reverse Transcriptase-Polymerase Chain Reaction

Quantitative real-time PCR was performed as described previously (Wang et al. 2020a; Wang et al. 2020b). In brief, total RNA from tissue or cells were isolated with TRIzol (#DP424, Tiangen Biotech Co. Ltd., Beijing, China). First-strand cDNA was synthesized from 1.5 μg of RNA using reverse transcriptase kits (ThermoFisher Scientific, Waltham, MA) according to the manufacturer’s instructions. After cDNA synthesis, the expressions of indicated genes were estimated by real-time PCR using the SGExcel FastSYBR Mixture (#B532955-0005, Sangon Biotech Co., Ltd., Shanghai, China) on Roche LightCycler^R^ 480 Quantitative PCR System (Indianapolis, United States). The PCR results of GAPDH served as internal controls. The primers used for PCR are listed in Table 1.

**TABLE 1:** The primers used in this study for real time PCR.

Description	Sense primer (5′→3′)	Antisense primer (5′→3′)
*Fasn*	GGA​GGT​GGT​GAT​AGC​CGG​TAT	TGG​GTA​ATC​CAT​AGA​GCC​CAG
*Scd1*	TTC​TTG​CGA​TAC​ACT​CTG​GTG​C	CGG​GAT​TGA​ATG​TTC​TTG​TCG​T
*Acc1*	CTC​CCG​ATT​CAT​AAT​TGG​GTC​TG	TCG​ACC​TTG​TTT​TAC​TAG​GTG​C
*Hmgcr*	TGA​CCT​TTC​TAG​AGC​GAG​TGC​AT	CAC​GAG​CTA​TAT​TTT​CCC​TTA​CTT​CA
*Hmgcs*	AGA​GAG​CGA​TGC​AGG​AAA​CTT	AAG​GAT​GCC​CAC​ATC​TTT​TGG
*Gapdh*	TGA​GGC​CGG​TGC​TGA​GTA​TGT	CAG​TCT​TCT​GGG​TGG​CAG​TGA​T

### Immunoblotting

Immunoblotting was performed as described previously (Wang et al., 2020a). In brief, liver tissue or cells were extracted with 1 × sodium dodecyl sulfate (SDS). A total of 25 μg protein was loaded into a 10% SDS-PAGE gel and transferred onto a polyvinylidene fluoride (PVDF) membrane. Primary antibodies were incubated overnight, and the secondary antibodies (Cell Signaling Technology, 1:3000) were added onto the membrane. Immunoreactive bands were visualized using enhanced chemiluminescence reagents (#180-501, Tanon Biotechnology, Shanghai, China). Chemiluminescence was determined using Tanon 4200SF system (Tanon Biotechnology, Shanghai, China).

### Statistical Analysis

Data is presented as mean ± SD. Statistical analyses were performed using GraphPad Prism 7.0 (GraphPad Software, La Jolla California USA). Differences between the groups were analyzed using Student’s t-test or one-way ANOVA followed by Dunnett’s multiple comparisons test. Significance thresholds were *p* < 0.05.

## Results

### Lim Ameliorates Metabolic Disorder in HFD Fed Mice

To explore the effect of Lim on metabolic disorder, we built up our animal model by feeding mice high fat diet (Figure 1B). Firstly, we took thorough examination of metabolic parameters in these mice. There was no difference in food intake between control and Lim treated groups no matter what kind of diet was fed (Figure 1C). We observed significant body weight loss in Lim treated mice fed with HFD, and there was no obvious difference in body weights in mice fed with chow diet with or without Lim administration (Figures 1D,E). Lim reduced the amount of epididymal and subcutaneous white adipose tissue in HFD fed mice (Figure 1F). Moreover, Lim treatment improved systematic insulin resistance and lowered serum lipids in HFD-fed mice (Supplementary Figures S1, S2). Of note, the increasing of liver weight induced by HFD feeding was also ameliorated by Lim treatment (Figure 1G).

### Lim Improves Liver Function in HFD Fed Mice

**FIGURE 2:**
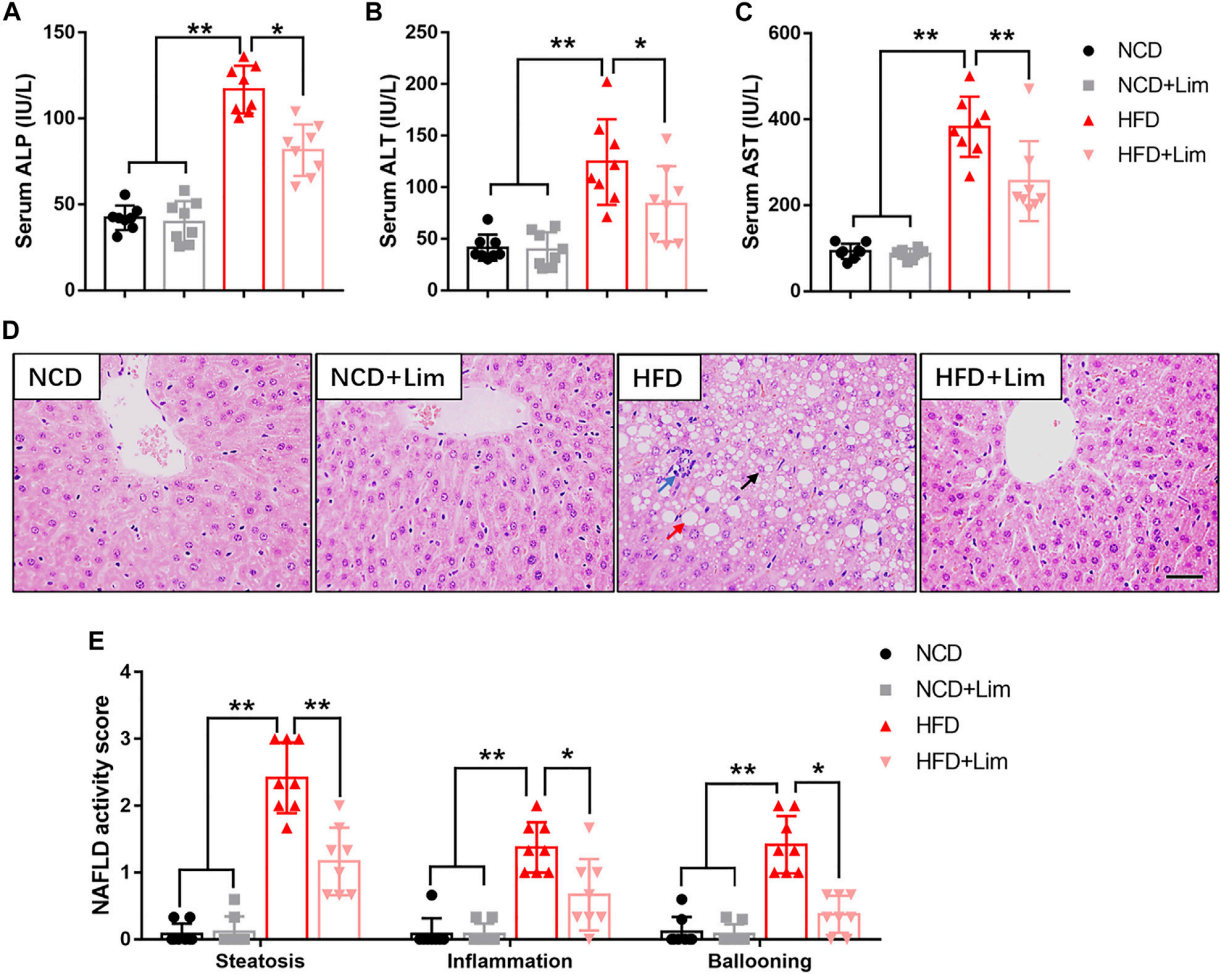
Effects of Lim on liver function in HFD fed mice. **(A)** Serum ALP (Alkaline phosphatase) levels; **(B)** Serum ALT (Alanine Aminotransferase) levels; **(C)** Serum AST (Aspartate Aminotransferase) levels. **(D)** The representative images of H&E staining in livers from each group. Scale bar = 300 μm. Black arrow denotes macrovesicular steatosis; red arrow denotes hepatocellular ballooning; blue arrow denotes lobular inflammation. **(E)** Quantification of NAS (NAFLD activity score) based on 3 histologic features (steatosis 0-3, inflammation 0-3, hepatocellular ballooning 0–2) in H&E-stained liver sections. Data were expressed as the mean ± SD (*n* = 8). ^*^ *p* < 0.05, ^**^ *p* < 0.01.

Next, we focused on the effect of Lim on liver function under the condition of HFD feeding. As shown in Figures 2A–C, Lim significantly improved liver function in HFD fed mice revealed by decreased levels of ALP, ALT and AST in serum. The pathologic change of livers was determined by H&E staining and semi-quantitative analysis. We found that Lim obviously ameliorated hepatic steatosis and the infiltration of inflammatory cells in HFD fed mice (Figures 2D,E).

### Lim Reduces Hepatic Lipid Accumulation in HFD Fed Mice

**FIGURE 3:**
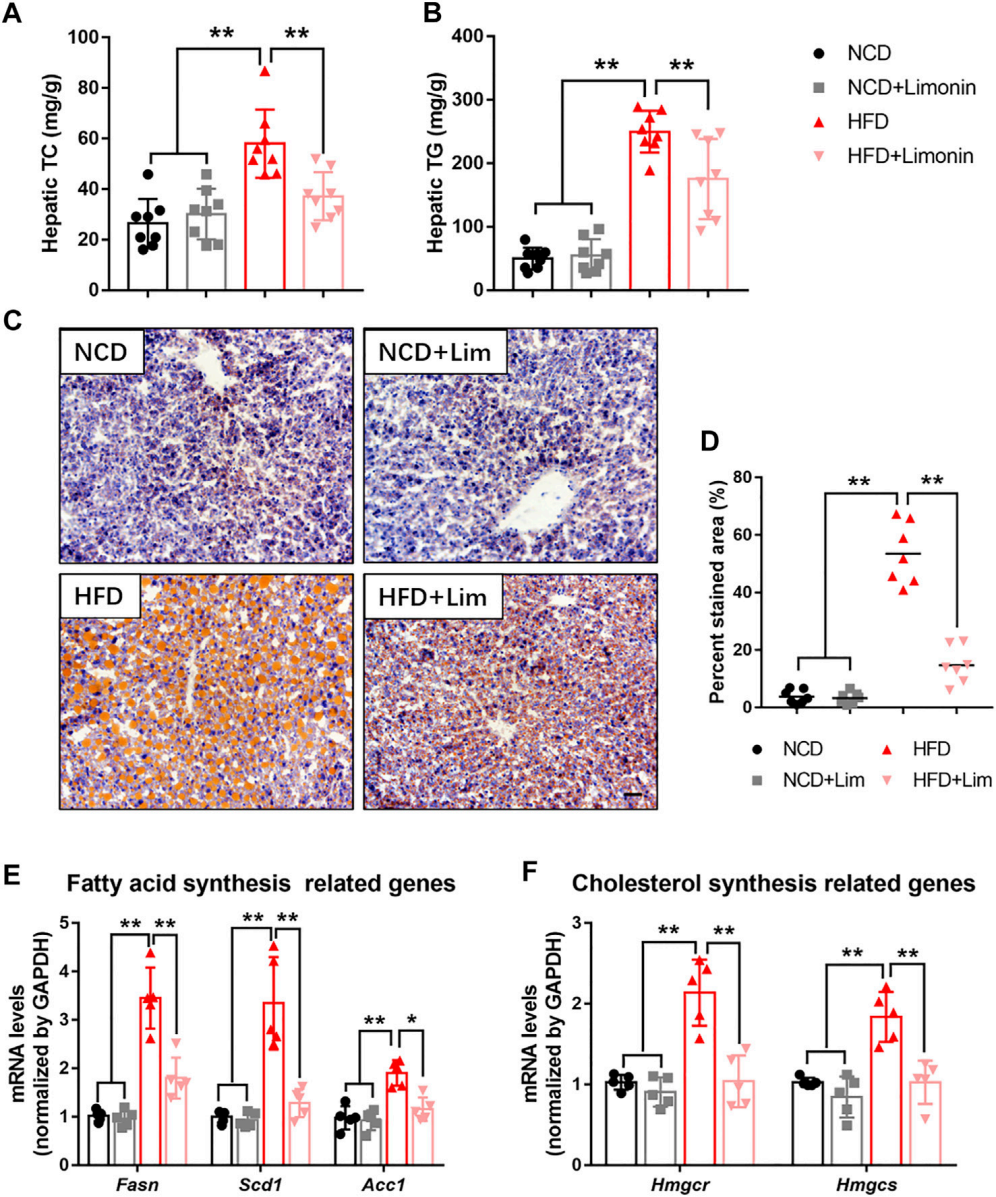
Lim suppresses HFD-induced hepatic lipid accumulation in mice. **(A)** Hepatic TC levels. **(B)** Hepatic TG levels. **(C)** The representative images of Oil Red O staining in livers from each group. Scale bar = 300 μm. **(D)** The quantification of Oil Red O-stained areas was shown. Data were expressed as the mean ± SD (*n* = 8). The mRNA levels of genes related to fatty acid synthesis **(E)** and cholesterol synthesis **(F)** were determined by real-time PCR. Data were expressed as the mean ± SD (*n* = 5). ^*^ *p* < 0.05, ^**^ *p* < 0.01.

To further characterize the effect of Lim on hepatic lipid accumulation, we evaluated the lipid content of mouse liver by several approaches. We found that Lim treatment significantly reduced the content of total cholesterol (TC) and triglyceride (TG) in the livers of mice fed a high-fat diet (Figures 3A,B). The amount of hepatic neutral lipids determined by Oil Red O staining was also decreased in HFD induced mice after Lim administration (Figures 3C,D). Furthermore, the mRNA expression of genes associated with fatty acid synthesis (*Fasn*, *Scd1* and *Acc1*) and cholesterol synthesis (*Hmgcr* and *Hmgcs*) was down-regulated by Lim in the livers of HFD fed mice (Figures 3E,F).

### Lim Activates AMPK *in vitro* and *in vivo*

**FIGURE 4:**
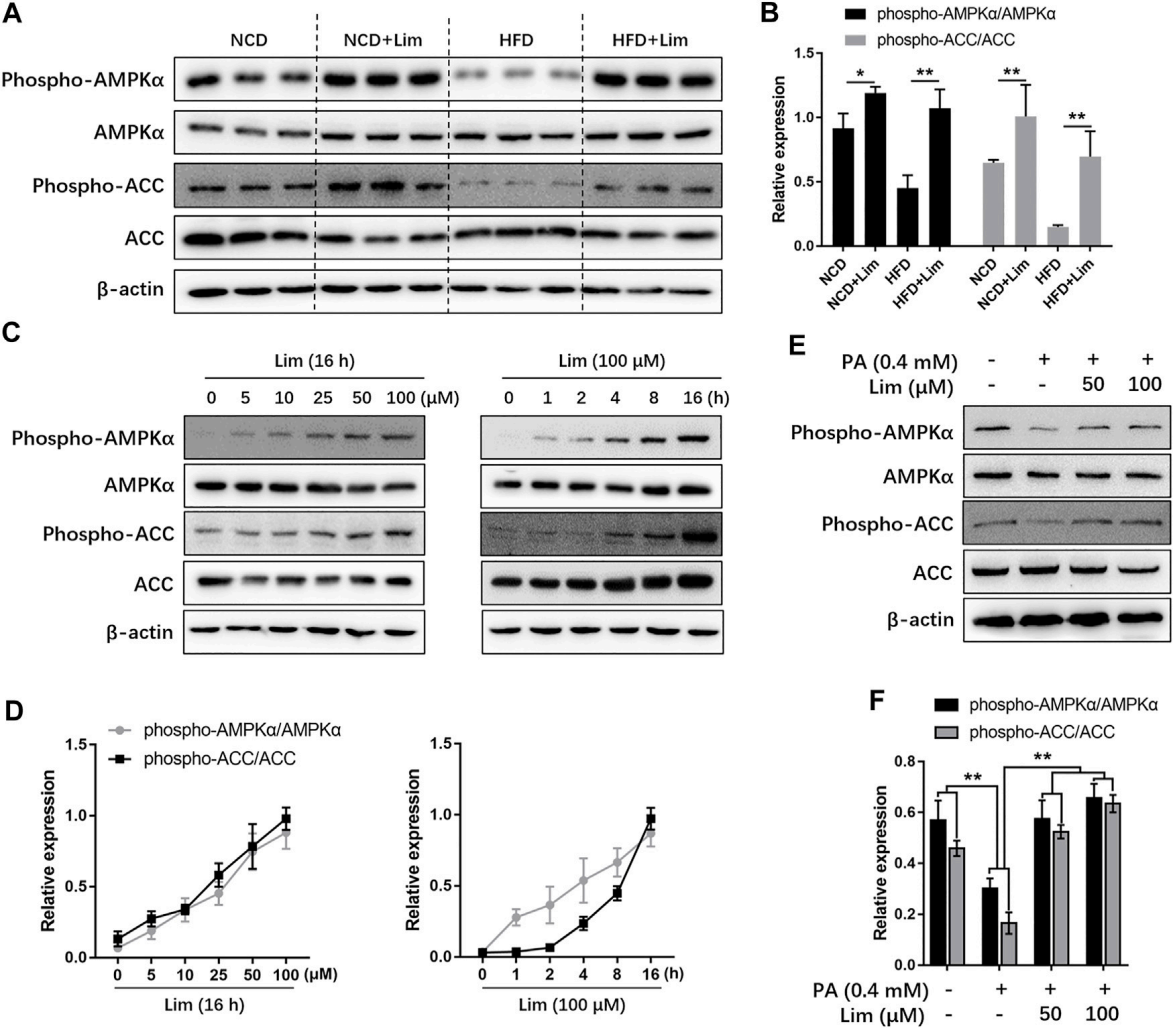
Lim increases AMPK activity *in vitro* and *in vivo*. **(A)** The protein level of phospho-AMPK, AMPK, phospho-ACC and ACC in livers of HFD-induced mice were assessed by Western blot analysis. **(C)** Lim increased AMPK activity in a dose and time-dependent manner in AML12 cells. **(E)** The AML12 cells were treated with DMSO, 0.4 mM palmitic acid (PA), 0.4 mM PA + 50 μM Lim or 0.4 mM PA + 100 μM Lim for 16 h respectively, after starving in serum-free DMEM for 24 h. The amount of phospho-AMPK, AMPK, phospho-ACC and ACC was determined by Western blotting. **(B,D,F)** The intensity of bands for phospho-AMPK and phospho-ACC was normalized to total AMPK and ACC, respectively. Data were expressed as the mean ± SD (*n* = 3). ^*^ *p* < 0.05, ^**^ *p* < 0.01.

AMPK is a master regulator to keep metabolic balance in liver tissue. Previous study reported that triterpenoids can activate AMPK (Tan et al., 2008). Lim is the first tetranortriterpenoid obtained from citrus bitter principles (Roy and Saraf 2006). Therefore, we wondered if Lim could increase AMPK activity. We found that HFD feeding inhibited AMPK phosphorylation in mouse livers, while Lim treatment greatly enhanced AMPK phosphorylation, especially in HFD mice. Meanwhile, the phosphorylation of ACC, a well-known substate of AMPK, was also increased by Lim under the condition of HFD feeding (Figures 4A,B). To determine if the activation of AMPK is a direct action of Lim on hepatocytes in the livers, or due to its indirect effect on whole body metabolism, we treated murine hepatic cell line AML12 with Lim in different concentrations or time. As shown in Figures 4C,D, Lim induced AMPK activation and ACC phosphorylation in a dose- and time-dependent manner. To mimic HFD feeding *in vitro*, we treated AML12 cells with palmitic acid (PA). Similar with the observation *in vivo*, PA challenge decreased the phosphorylation of both AMPK and ACC, while Lim treatment re-elevated both of them in PA challenged cells (Figures 4E,F).

### Lim Ameliorates Lipid Accumulation *via* AMPK Activation

**FIGURE 5:**
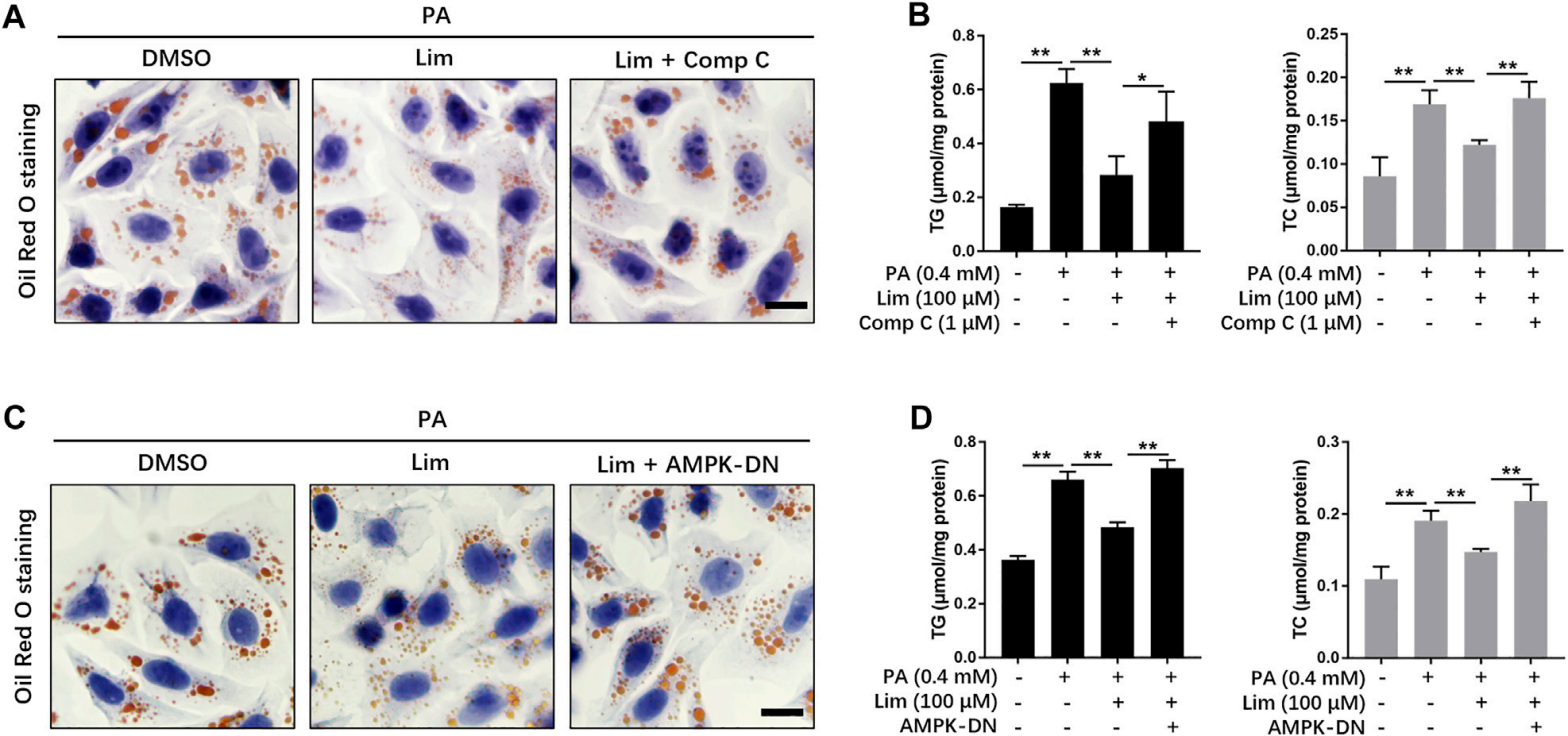
The role of AMPK in Lim derived reduction of lipid accumulation in hepatocytes. **(A)** The AML12 cells were treated with 0.4 mM PA, 0.4 mM PA + 100 μM Lim or 0.4 mM PA + 100 μM Lim +1 μM Comp C for 16 h respectively, after starving in serum-free DMEM for 24 h. The representative images of Oil Red O staining in cells. Scale bar = 300 μm. **(B)** The AML12 cells were treated with DMSO, 0.4 mM PA, 0.4 mM PA + 100 μM Lim or 0.4 mM PA + 100 μM Lim +1 μM Comp C for 16 h respectively, after starving in serum-free DMEM for 24 h. The intracellular levels of TG and TC were determined by a colorimetric enzymatic assay. **(C,D)** Dominant negative AMPK expressing plasmid (AMPK-DN) was introduced into AML12 before treatment with PA and Lim. The lipid-lowering effect of Lim was abolished by AMPK-DN in AML12 cells treated with PA, as revealed by Oil Red O staining and the measurement of intracellular TG and TC. Data were expressed as the mean ± SD (*n* = 3). ^*^ *p* < 0.05, ^**^ *p* < 0.01.

To investigate whether the activation of AMPK is required for the reductive effect of Lim on lipid accumulation, we suppressed AMPK activity *in vitro* by Dorsomorphin (Compound C) or the expression of AMPK dominant negative plasmid. As shown in Figure 5A and Supplementary Figure S3, Lim significantly lowered PA induced lipid accumulation in AML12 cells, while Compound C almost completely abolished the effect. Similar with the result from Oil Red O staining, the measurement of TG and TC also showed that the administration of Compound C reversed Lim induced decrease of intracellular lipids (Figure 5B). In addition, Inhibition of AMPK activity by the expression of dominant negative AMPK plasmid also greatly reversed the repressive effect of Lim on lipid accumulation (Figures 5C,D).

### Lim Suppresses Fat Accumulation in *Caenorhabditis elegans* Through AMPK

**FIGURE 6:**
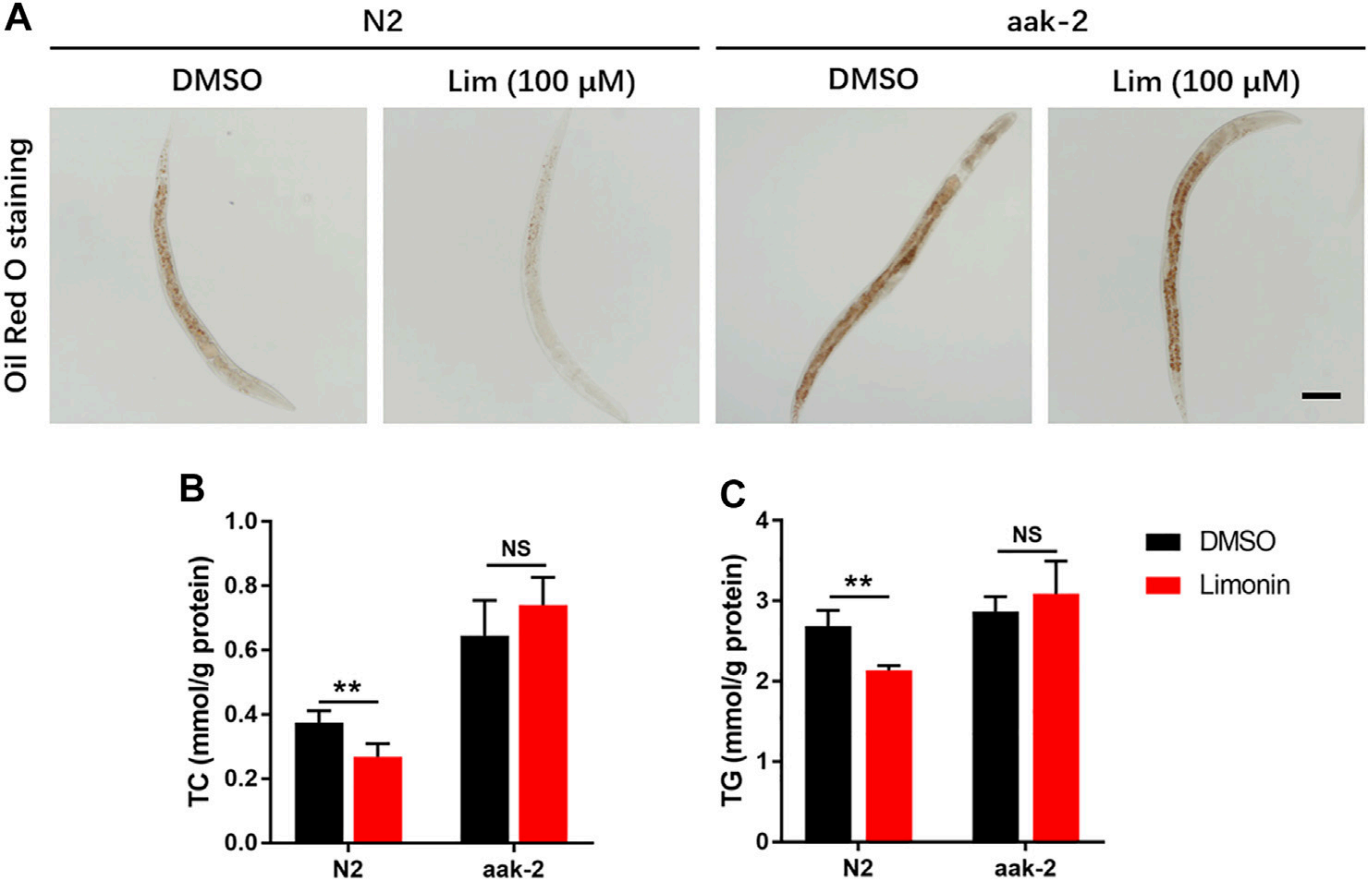
The inhibition of fat deposition by Lim in *Caenorhabditis elegans* requires AMPK. N2 (wild-type) and aak-2 (AMPK knockout) worms were treated with or without Lim (100 μM) for 7 days. **(A)** The representative images of Oil Red O staining in worms. Scale bar = 300 μm. **(B,C)** The TC and TG levels in worms were determined by a colorimetric enzymatic assay. Data were expressed as the mean ± SD (*n* = 6). ^**^ *p* < 0.01; NS, no significance.

To further confirm AMPK activation is also necessary to Lim induced reduction of lipid accumulation *in vivo*, we used genetic model from *Caenorhabditis elegans.* Lim administration reduced the amount of lipid droplets in N2 worms determined by Oil Red O staining and TC/TG measurement. In aak-2 worms, knockout of AMPK completely abrogated Lim induced suppression of fat accumulation (Figures 6A–C).

### Lim Activates AMPK Through Inhibition of ATP Generation

**FIGURE 7:**
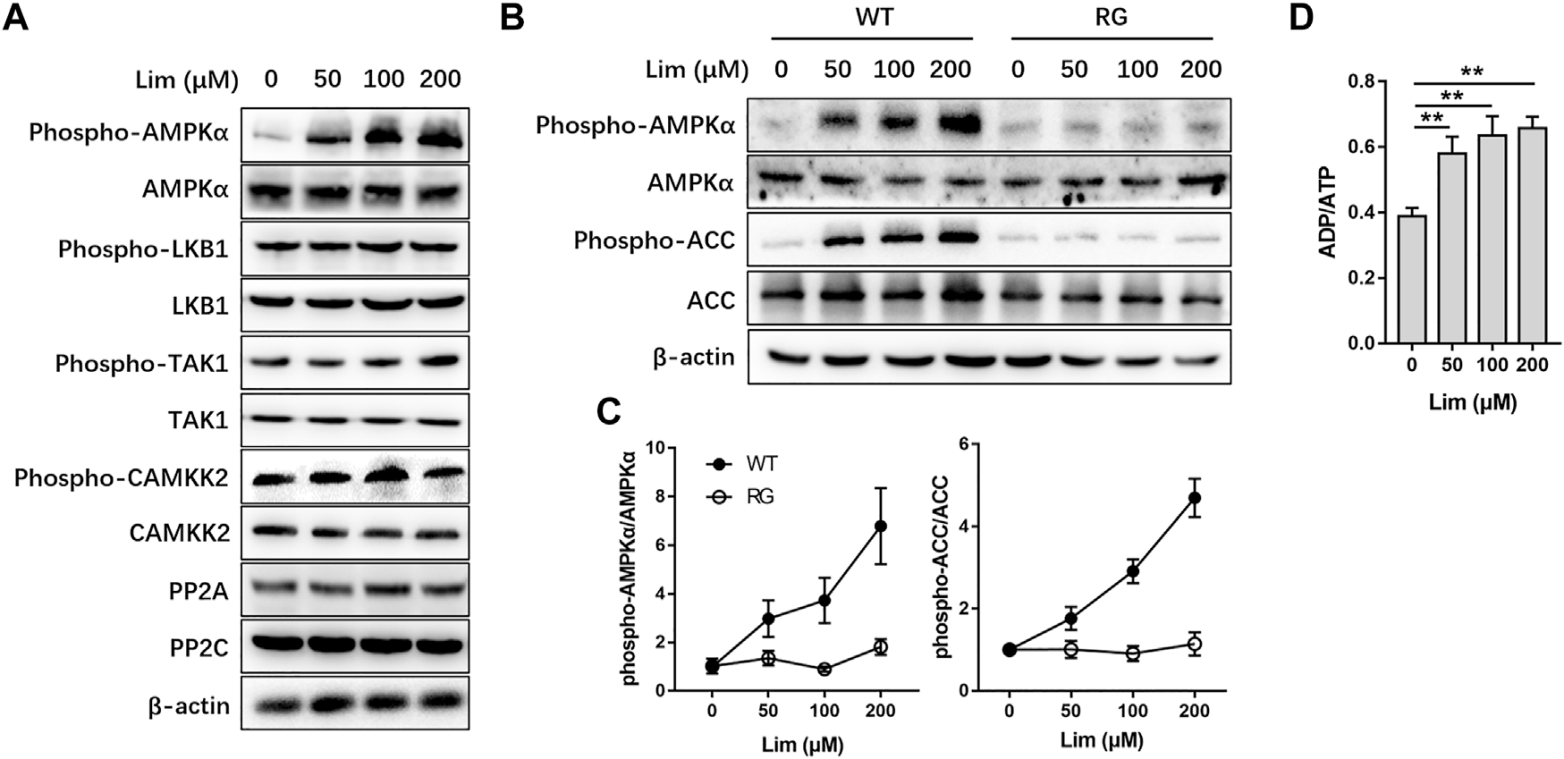
The activation of AMPK by Lim was adenine nucleotide dependent. HepG2 cells were treated with different concentrations of Lim for 4 h. **(A)** The abundance or activation of protein kinases and phosphatases upstream of AMPK were examined by Western blotting. **(B)** HepG2 cells were transduced with wild-type (WT) or R531G (RG) AMPKγ2 before Lim treatment. The activation of AMPK and the phosphorylation of ACC were examined by Western blotting. **(C)** The intensity of bands for phospho-AMPK and phospho-ACC was normalized to total AMPK and ACC, respectively. **(D)** Lim increased the intracellular ratio of ADP and ATP in HepG2. Data were expressed as the mean ± SD (*n* = 3). ^**^ *p* < 0.01.

Next, we tried to find out how Lim activated AMPK. The phosphorylation of Thr172 and the binding of AMP are considered as two major ways to activate AMPK (Herzig and Shaw, 2018). There are several alternative upstream activators and negative regulators in mammals for the regulation of AMPK activity (Jian et al., 2020). We found that neither the upstream kinases, including liver kinase B1 (LKB1), calcium/calmodulin-dependent protein kinase 2 (CAMKK2), and transforming growth factor *β* activated kinase 1 (TAK1), nor the negative regulators such as protein phosphatase 2A (PP2A) and PP2C were significantly affected by Lim treatment (Figure 7A). To determine whether AMPK activation by Lim was AMP dependent, we performed the assay firstly introduced by Simon A. Hawley on *Cell Metabolism* (Hawley et al., 2010). As shown in Figures 7B,C, Lim induced AMPK activation and ACC phosphorylation in a dose-manner in WT but not in RG cells, suggesting Lim treatment inhibited ATP production. Indeed, Lim significantly increased ADP/ATP ratio measured by bioluminescent assay kit in parental 293T cells (Figure 7D).

### Lim Suppresses the Transcriptional Activity of SREBP1/2 Through AMPK

**FIGURE 8:**
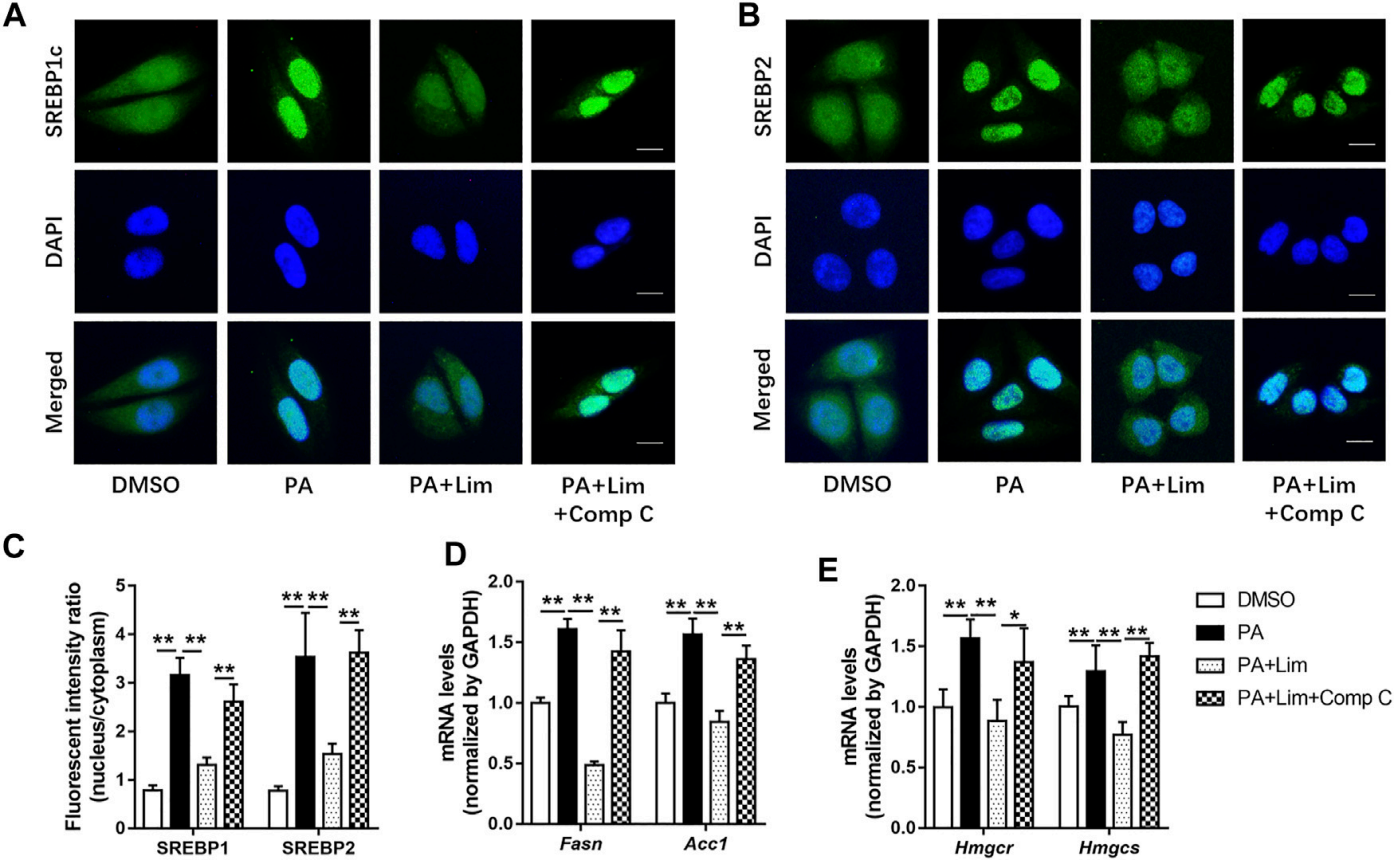
Lim inhibits the transcriptional activity of SREBP1 and SREBP2 through AMPK in hepatocytes. The AML12 cells were treated with DMSO, 0.4 mM palmitic acid (PA), 0.4 mM PA + 100 μM Lim or 0.4 mM PA + 100 μM Lim +1 μM Comp C for 16 h respectively, after starving in serum-free DMEM for 24 h **(A,B)** The representative images of SREBP1 and SREBP2 immunofluorescent staining in cells. Scale bar = 300 μm. **(C)** Quantification of the fluorescent intensity of SREBP1 and SREBP2 in the nuclei relative to that of the cytoplasm. **(D,E)** The mRNA levels of *Fasn*, *Acc1* and *Hmgcr*, *Hmgcs* genes were evaluated using RT-PCR. Values were expressed as mean ± SD (*n* = 3). ^*^ *p* < 0.05, ^**^ *p* < 0.01.

Since the expression of fatty acid and cholesterol synthesis genes were down-regulated by Lim in the livers of HFD fed mice, we wondered whether Lim inhibited the expression of these genes by activating AMPK. To test our hypothesis, we determined the intracellular localization of SREBP1c and SREBP2, two most crucial transcriptional factors for lipid metabolism gene expression, after PA induction. As shown in Figures 8A,B, PA induced nuclear translocation of SREBP1c and SREBP2, while Lim treatment decreased the gathering of these two proteins in nuclei. Inhibition of AMPK by Compound C enhanced the nuclear translocation of SREBP1c and SREBP2 in Lim treated hepatocytes (Figures 8A–C). In addition, we examined the expression of downstream targets of SREBP1c and SREBP2. The administration of Compound C significantly re-elevated the expression of *Fasn* and *Acc1* regulated by SREBP1c, and the expression of *Hmgcr* and *Hmgcs* regulated by SREBP2 in Lim treated AML12 cells (Figures 8D,E).

## Discussion

Here, we identify, for the first time, that limonin inhibits hepatic lipid accumulation in HFD fed mice via the activation of AMPK. Limonoids are highly oxygenated triterpenoid compounds. Limonin, nomilin and obacunone are the major limonoids in Citrus (Gualdani et al., 2016). In 2011, Eri Ono’s publication stated that nomilin and obacunone can activate TGR5, a bile acid membrane receptor (Ono et al., 2011). The activation of TGR5 by bile acids increases energy expenditure and attenuates diet-induced obesity in mice (Thomas et al., 2009). Eri Ono suggested that the anti-obesity and anti-hyperglycemic effects by nomilin was through activating TGR5. Furthermore, they pointed out that limonin was not a TGR5 agonist (Ono et al., 2011). Despite some studies demonstrated the effect of limonin on reducing inflammation and oxidative stress, few reported the function of limonin in the regulation of glucose and lipid metabolism, as its incapability of activating TGR5. The current study uncovered the significant role of limonin in metabolic regulation. During our previous studies, we investigated the effects of several active compounds in the citrus aurantium on NAFLD (Bai et al., 2019; Wang et al. 2020a; Wang et al. 2021a). Using cell based system, we screened natural products with regulatory effect on AMPK activity. We found that limonin can activate AMPK *in vivo* and *in vitro*. As we known, there has been no report on the activation of AMPK by limonin so far. The key regulatory roles of AMPK on glucose and lipid homeostasis further support observation that limonin can effectively ameliorate metabolic disorders including NAFLD.

A wide variety of drugs and xenobiotics has been found to activate AMPK, for example, antidiabetic drugs like metformin, phenformin and thiazolidinediones, and natural products derived from traditional medicines or foods such as berberine, quercetin, resveratrol and epigallocatechin gallate (Hawley et al., 2010). However, the underlying mechanisms by which AMPK is activated are remain unclear in many cases. Adenine nucleotides are compartmentalized between the mitochondria and cytoplasm and their diffusion is limited, they may not be uniformly distributed within the cytoplasm. The AMPK is extremely sensitive to small changes in AMP. Subtle changes in subcellular nucleotides that may not be detectable by measuring total cellular levels would be enough to influence the activity of AMPK. The measurement of cellular contents of AMP and ADP in cell extracts will lose the information of their effects of compartmentation. Therefore, while an agent like metformin may activate AMPK without producing a detectable change in AMP, this does not prove the effect is AMP independent (Hawley et al., 2010). To determine whether or not activation of AMPK by different agents was AMP dependent, Simon A. Hawley et al. (2010) developed a test. The mutation of AMPKγ2 subunit, R531G, causes a severe loss of binding by AMP and ATP, thus generating an AMP-insensitive AMPK complex. They expressed wild-type or R531G mutant γ2 subunit in cells to replace the endogenous γ1 subunit in the assay. This method was widely adopted by later researches, and was carried out in our current study. We used oligomycin and A769662 as positive controls to validate the effectiveness of the test (Supplementary Figure S4). Using this system, we found that Lim activated AMPK through inhibition of cellular energy metabolism and increasing ADP: ATP ratio. However, more investigation is needed in future to elaborate whether Lim increased AMPK activity through initiating/inhibiting membrane receptor associated signal pathways, uncoupling the respiratory oxidation and ATP generation in the mitochondria, or interfering glycolysis.

At the beginning of the current study, we examined the changes of systemic metabolism in mice with or without limonin treatment. Although the remission of liver pathological changes is the focus of our attention, it is obvious that limonin has a significant impact on systemic metabolic indicators, including body weight, body fat, blood sugar, blood lipids and insulin sensitivity. These indicators are affected by diverse organs such as liver, adipose tissue, skeletal muscle as well as other factors like neuroendocrine and intestinal flora. Conversely, changes in these indicators also affect the metabolic status of liver, adipose tissue, skeletal muscle and other organs. Through cell based assays *in vitro*, we determined that limonin had a direct effect on hepatic cells. Even though, we can’t rule out the possibility that limonin relieved hepatic steatosis and inflammation in mice induced by high-fat diet through its joint action on the liver and other tissues such as adipose tissue. In the body, limonin may first act on the liver, and affect the metabolism of adipose tissue through secretory factors like hepatokines, thereby improving systemic metabolism. It may also act directly on adipose tissue, lowering blood lipids, thereby alleviating hepatic steatosis. This effect superimposed with its direct effect on the liver, resulting in the overall influence we observed. These speculations warrant more investigations in future.

## Conclusion

In summary, we found that limonin activated AMPK in hepatocytes and attenuated hepatic lipid accumulation. These findings suggest limonin as potential therapeutics for MAFLD and warrant more detailed investigation for its underlying mechanisms in future.

## Data Availability

The original contributions presented in the study are included in the article/Supplementary Material, further inquiries can be directed to the corresponding author.

## References

[B1] Bai Y. F. Wang S. W. Wang X. X. Weng Y. Y. Fan X. Y. Sheng H. (2019). The Flavonoid-Rich Quzhou Fructus Aurantii Extract Modulates Gut Microbiota and Prevents Obesity in High-Fat Diet-Fed Mice. Nutr. Diabetes 9 (1), 30. 10.1038/s41387-019-0097-6 31645541 PMC6811639

[B2] Bhathena S. J. Velasquez M. T. (2002). Beneficial Role of Dietary Phytoestrogens in Obesity and Diabetes. Am. J. Clin. Nutr. 76 (6), 1191–1201. 10.1093/ajcn/76.6.1191 12450882

[B3] Chidambara Murthy K. N. Jayaprakasha G. K. Safe S. Patil B. S. (2021). Citrus Limonoids Induce Apoptosis and Inhibit the Proliferation of Pancreatic Cancer Cells. Food Funct. 12 (3), 1111–1120. 10.1039/d0fo02740e 33427831

[B4] Eslam M. Sanyal A. J. George J. International Consensus P. (2020). MAFLD: A Consensus-Driven Proposed Nomenclature for Metabolic Associated Fatty Liver Disease. Gastroenterology 158 (7), 1999–e1. 10.1053/j.gastro.2019.11.312 32044314

[B5] Fan S. Zhang C. Luo T. Wang J. Tang Y. Chen Z. (2019). Limonin: A Review of Its Pharmacology, Toxicity, and Pharmacokinetics. Molecules 24 (20). 10.3390/molecules24203679 PMC6832453 31614806

[B6] Garcia D. Hellberg K. Chaix A. Wallace M. Herzig S. Badur M. G. (2019). Genetic Liver-specific AMPK Activation Protects against Diet-Induced Obesity and NAFLD. Cell Rep 26 (1), 192–e6. 10.1016/j.celrep.2018.12.036 30605676 PMC6344045

[B7] Garcia D. Shaw R. J. (2017). AMPK: Mechanisms of Cellular Energy Sensing and Restoration of Metabolic Balance. Mol. Cell 66 (6), 789–800. 10.1016/j.molcel.2017.05.032 28622524 PMC5553560

[B8] Gu M. Sun J. Qi C. Cai X. Goulette T. Song M. (2019). The Gastrointestinal Fate of Limonin and its Effect on Gut Microbiota in Mice. Food Funct. 10 (9), 5521–5530. 10.1039/c9fo01274e 31418448 PMC6751002

[B9] Gualdani R. Cavalluzzi M. M. Lentini G. Habtemariam S. (2016). The Chemistry and Pharmacology of Citrus Limonoids. Molecules 21 (11), 1530. 10.3390/molecules21111530 PMC6273274 27845763

[B10] Hawley S. A. Ross F. A. Chevtzoff C. Green K. A. Evans A. Fogarty S. (2010). Use of Cells Expressing Gamma Subunit Variants to Identify Diverse Mechanisms of AMPK Activation. Cell Metab 11 (6), 554–565. 10.1016/j.cmet.2010.04.001 20519126 PMC2935965

[B11] Heeren J. Scheja L. (2021). Metabolic-associated Fatty Liver Disease and Lipoprotein Metabolism. Mol. Metab. 50, 101238. 10.1016/j.molmet.2021.101238 33892169 PMC8324684

[B12] Herzig S. Shaw R. J. (2018). AMPK: Guardian of Metabolism and Mitochondrial Homeostasis. Nat. Rev. Mol. Cell Biol 19 (2), 121–135. 10.1038/nrm.2017.95 28974774 PMC5780224

[B13] Jian C. Fu J. Cheng X. Shen L. J. Ji Y. X. Wang X. (2020). Low-Dose Sorafenib Acts as a Mitochondrial Uncoupler and Ameliorates Nonalcoholic Steatohepatitis. Cell Metab 31 (5), 892–e11. e811. 10.1016/j.cmet.2020.04.011 32375062 PMC9375823

[B14] Li Y. Xu S. Mihaylova M. M. Zheng B. Hou X. Jiang B. (2011). AMPK Phosphorylates and Inhibits SREBP Activity to Attenuate Hepatic Steatosis and Atherosclerosis in Diet-Induced Insulin-Resistant Mice. Cell Metab 13 (4), 376–388. 10.1016/j.cmet.2011.03.009 21459323 PMC3086578

[B15] Ono E. Inoue J. Hashidume T. Shimizu M. Sato R. (2011). Anti-obesity and Anti-hyperglycemic Effects of the Dietary Citrus Limonoid Nomilin in Mice Fed a High-Fat Diet. Biochem. Biophys. Res. Commun. 410 (3), 677–681. 10.1016/j.bbrc.2011.06.055 21693102

[B16] Ooi G. J. Meikle P. J. Huynh K. Earnest A. Roberts S. K. Kemp W. (2021). Hepatic Lipidomic Remodeling in Severe Obesity Manifests with Steatosis and Does Not Evolve with Non-alcoholic Steatohepatitis. J. Hepatol. 75 (3), 524–535. 10.1016/j.jhep.2021.04.013 33887358

[B17] Roy A. Saraf S. (2006). Limonoids: Overview of Significant Bioactive Triterpenes Distributed in Plants Kingdom. Biol. Pharm. Bull. 29 (2), 191–201. 10.1248/bpb.29.191 16462017

[B18] Tan M. J. Ye J. M. Turner N. Hohnen-Behrens C. Ke C. Q. Tang C. P. (2008). Antidiabetic Activities of Triterpenoids Isolated from Bitter Melon Associated with Activation of the AMPK Pathway. Chem. Biol. 15 (3), 263–273. 10.1016/j.chembiol.2008.01.013 18355726

[B19] Tang H. Yu R. Liu S. Huwatibieke B. Li Z. Zhang W. (2016). Irisin Inhibits Hepatic Cholesterol Synthesis via AMPK-SREBP2 Signaling. EBioMedicine 6, 139–148. 10.1016/j.ebiom.2016.02.041 27211556 PMC4856751

[B20] Thomas C. Gioiello A. Noriega L. Strehle A. Oury J. Rizzo G. (2009). TGR5-mediated Bile Acid Sensing Controls Glucose Homeostasis. Cell Metab 10 (3), 167–177. 10.1016/j.cmet.2009.08.001 19723493 PMC2739652

[B21] Wang S. W. Bai Y. F. Weng Y. Y. Fan X. Y. Huang H. Zheng F. (2019). Cinobufacini Ameliorates Dextran Sulfate Sodium-Induced Colitis in Mice through Inhibiting M1 Macrophage Polarization. J. Pharmacol. Exp. Ther. 368 (3), 391–400. 10.1124/jpet.118.254516 30606760

[B22] Wang S. W. Lan T. Sheng H. Zheng F. Lei M. K. Wang L. X. (2021a). Nobiletin Alleviates Non-alcoholic Steatohepatitis in MCD-Induced Mice by Regulating Macrophage Polarization. Front. Physiol. 12, 687744. 10.3389/fphys.2021.687744 34093242 PMC8174844

[B23] Wang S. W. Sheng H. Bai Y. F. Weng Y. Y. Fan X. Y. Lou L. J. (2020a). Neohesperidin Enhances PGC-1α-Mediated Mitochondrial Biogenesis and Alleviates Hepatic Steatosis in High Fat Diet Fed Mice. Nutr. Diabetes 10 (1), 27. 10.1038/s41387-020-00130-3 32759940 PMC7406515

[B24] Wang S. W. Sheng H. Bai Y. F. Weng Y. Y. Fan X. Y. Zheng F. (2021b). Inhibition of Histone Acetyltransferase by Naringenin and Hesperetin Suppresses Txnip Expression and Protects Pancreatic β Cells in Diabetic Mice. Phytomedicine 88, 153454. 10.1016/j.phymed.2020.153454 33663922

[B25] Wang S. W. Wang W. Sheng H. Bai Y. F. Weng Y. Y. Fan X. Y. (2020b). Hesperetin, a SIRT1 Activator, Inhibits Hepatic Inflammation via AMPK/CREB Pathway. Int. Immunopharmacol 89 (Pt B), 107036. 10.1016/j.intimp.2020.107036 33068864

[B26] Wang S. W. Xu Y. Weng Y. Y. Fan X. Y. Bai Y. F. Zheng X. Y. (2018). Astilbin Ameliorates Cisplatin-Induced Nephrotoxicity through Reducing Oxidative Stress and Inflammation. Food Chem. Toxicol. 114, 227–236. 10.1016/j.fct.2018.02.041 29471006

[B27] Yang R. Song C. Chen J. Zhou L. Jiang X. Cao X. (2020). Limonin Ameliorates Acetaminophen-Induced Hepatotoxicity by Activating Nrf2 Antioxidative Pathway and Inhibiting NF-κB Inflammatory Response via Upregulating Sirt1. Phytomedicine 69, 153211. 10.1016/j.phymed.2020.153211 32259676

[B28] Yang R. Yu H. Chen J. Zhu J. Song C. Zhou L. (2021). Limonin Attenuates LPS-Induced Hepatotoxicity by Inhibiting Pyroptosis via NLRP3/Gasdermin D Signaling Pathway. J. Agric. Food Chem. 69 (3), 982–991. 10.1021/acs.jafc.0c06775 33427450

[B29] Yu J. Wang L. Walzem R. L. Miller E. G. Pike L. M. Patil B. S. (2005). Antioxidant Activity of Citrus Limonoids, Flavonoids, and Coumarins. J. Agric. Food Chem. 53 (6), 2009–2014. 10.1021/jf0484632 15769128

[B30] Zhao P. Sun X. Chaggan C. Liao Z. In Wong K. He F. (2020). An AMPK-Caspase-6 axis Controls Liver Damage in Nonalcoholic Steatohepatitis. Science 367 (6478), 652–660. 10.1126/science.aay0542 32029622 PMC8012106

[B31] Zhu C. Tabas I. Schwabe R. F. Pajvani U. B. (2021). Maladaptive Regeneration - the Reawakening of Developmental Pathways in NASH and Fibrosis. Nat. Rev. Gastroenterol. Hepatol. 18 (2), 131–142. 10.1038/s41575-020-00365-6 33051603 PMC7854502

