# Supplementary material for: Limonin, an AMPK Activator, Inhibits Hepatic Lipid Accumulation in High Fat Diet Fed Mice
Source: Front Pharmacol. 2022 Jan 24;13:833705. doi: 10.3389/fphar.2022.833705 (PMC8819594; doi:10.3389/fphar.2022.833705)
Supplement: Supplementary file 1 [file DataSheet3.docx]

Supplementary Figures


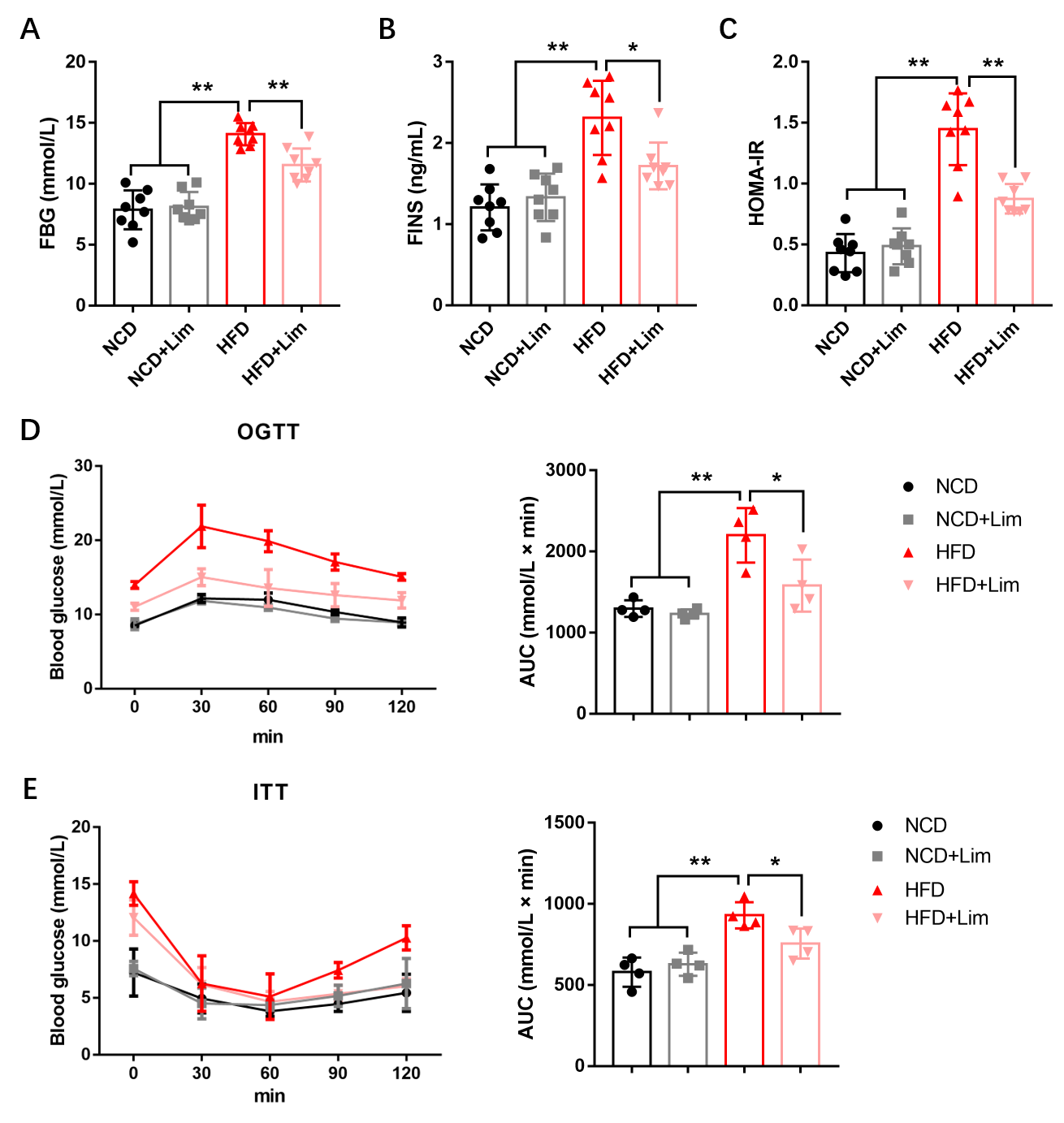


**Supplementary Fig. S1 The effect of Lim on systematic insulin resistance in HFD-fed mice.** C57BL/6 mice were fed either a chow diet as NCD or HFD for 10 weeks to induce fatty liver. Mice were treated with daily oral doses of Lim (50 mg/kg) from the second week of HFD diet feeding. Water was gavaged as control. **(A)** Fasting blood glucose (FBG) level in the last week. **(B)** Fasting insulin (FINS) level. **(C)** Homeostasis model assessment of insulin resistance (HOMA-IR) index. Data were expressed as the mean ± SD (n=8). **(D)** Oral glucose tolerance test (OGTT, 2 g/kg body weight, p.o.) in each group mice at week 10th. **(E)** Insulin tolerance test (ITT, 0.75 U/kg body weight, i.p.) in each group mice at week 10th. Data were expressed as the mean ± SD (n=4). ^*^*P* <0.05, ^**^*P* <0.01.

**
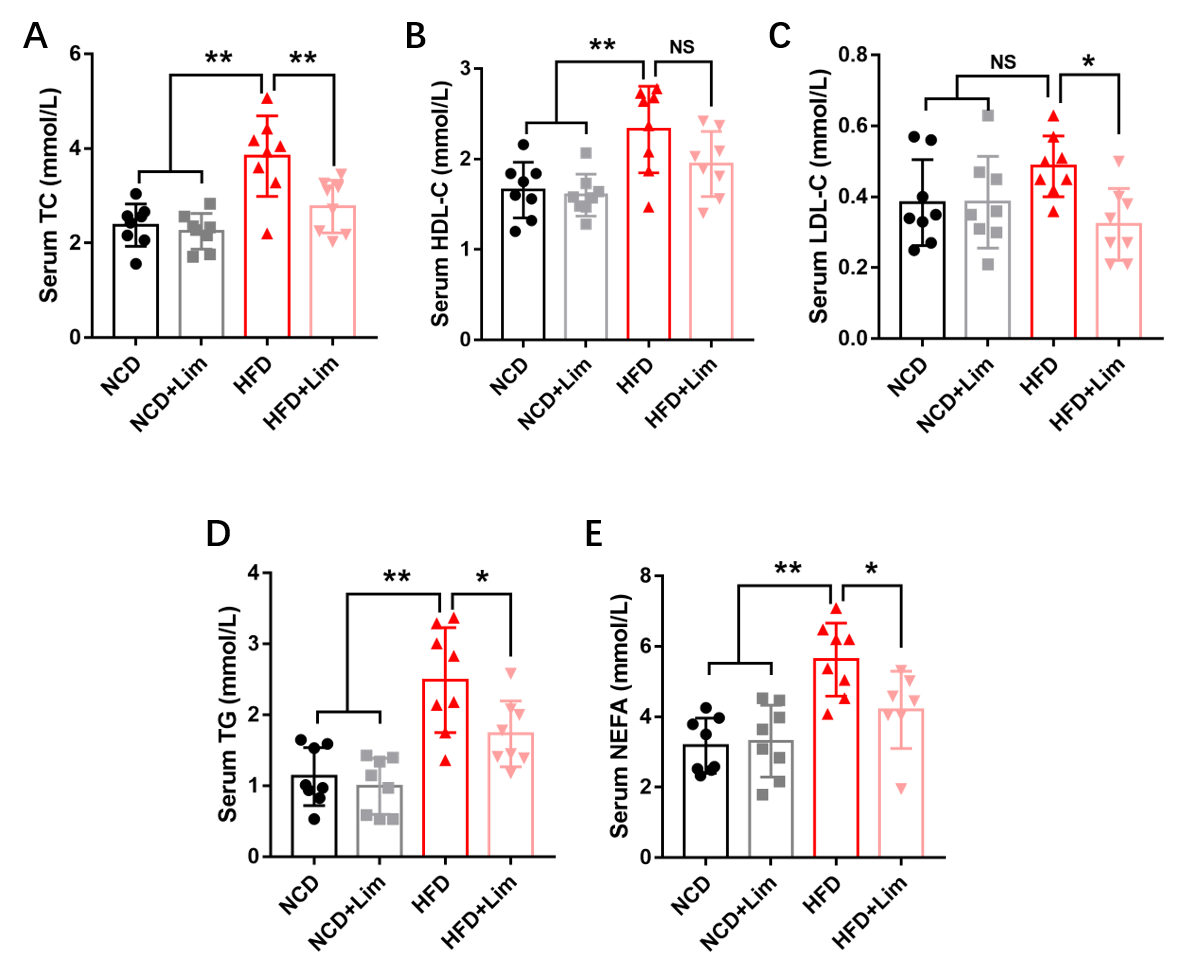
**

**Supplemental Fig. S2** **The effect of Lim on the levels of serum lipids in HFD-fed mice. (A)** Serum total cholesterol (TC) level; **(B)** Serum high-density lipoprotein cholesterol (HDL-C) level; **(C)** Serum low-density lipoprotein cholesterol (LDL-C) level; **(D)** Serum triglyceride (TG) level; **(E)** Serum nonesterified fatty acid (NEFA) level. Data were expressed as the mean ± SD (n=8). ^*^*P*<0.05, ^**^*P*<0.01; NS, no significance.


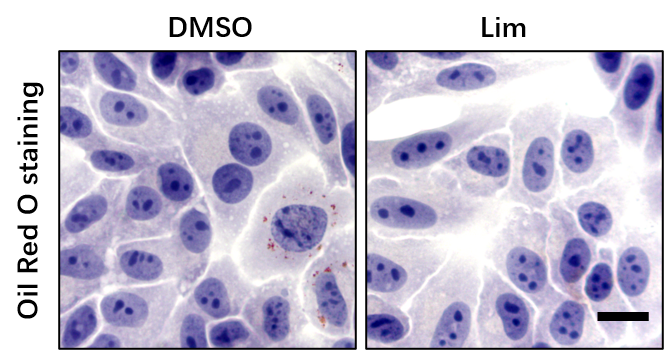


**Supplemental Fig. S3** **The effect of Lim on lipid accumulation in hepatocytes.** The AML12 cells were treated with DMSO or 100 μM Lim for 16 h, after starving in serum-free DMEM for 24 h. The representative images of Oil Red O staining in cells. Scale bar =300 μm.


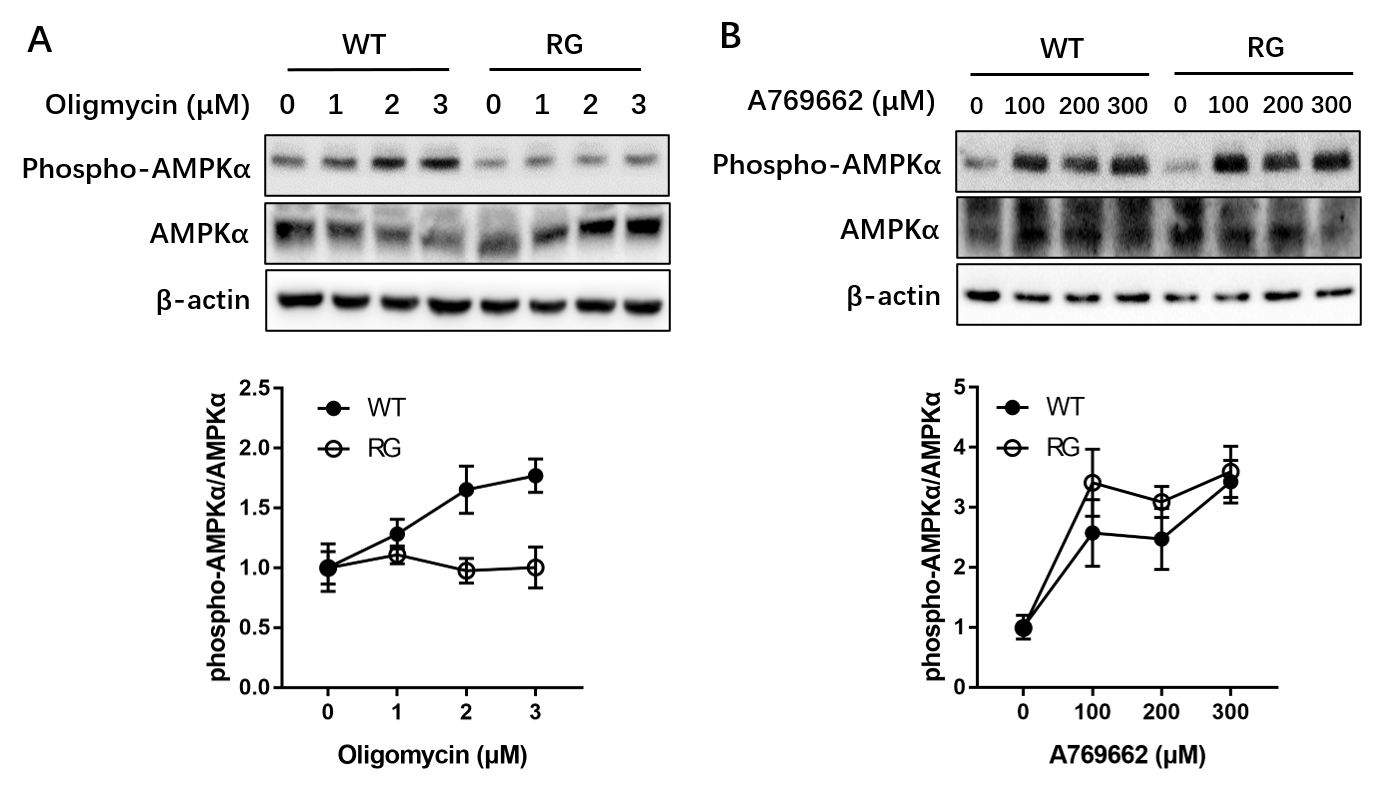


**Supplemental Fig. S4 Validation of the cells expressing AMPKγ2 subunit variant used to determine diverse mechanisms of AMPK activation.** HepG2 cells were transduced with wild-type (WT) or R531G (RG) AMPKγ2 and treated with different concentration of oligomycin **(A)** or A769662 **(B)**. The activation of AMPK and the phosphorylation of ACC were examined by Western blotting. Upper, representative result of Western blotting; lower, the ratio of phospho-AMPK and total AMPK. Data were expressed as the mean ± SD (n = 3).
