# Supplementary figures and images for: Limonin, an AMPK Activator, Inhibits Hepatic Lipid Accumulation in High Fat Diet Fed Mice
Source: Front Pharmacol. 2022 Jan 24;13:833705. doi: 10.3389/fphar.2022.833705 (PMC8819594; doi:10.3389/fphar.2022.833705)

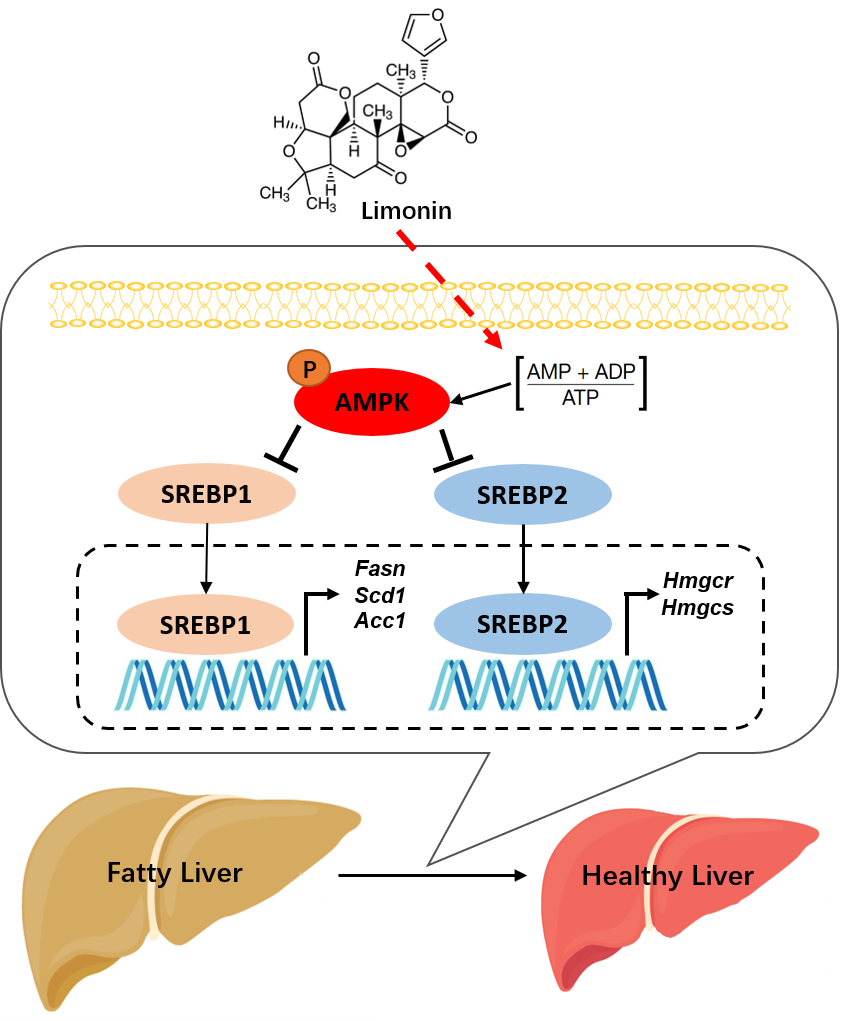

Supplement: Supplementary file 2 [file Image1.TIF]

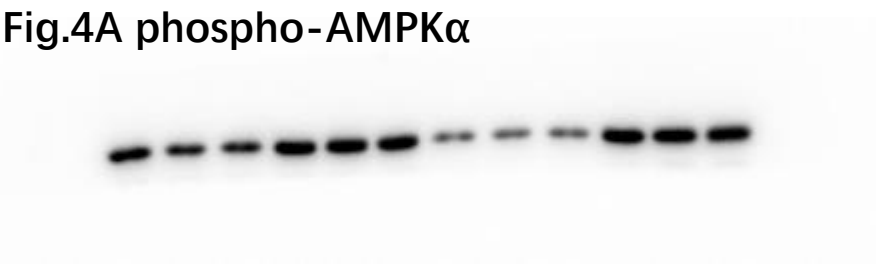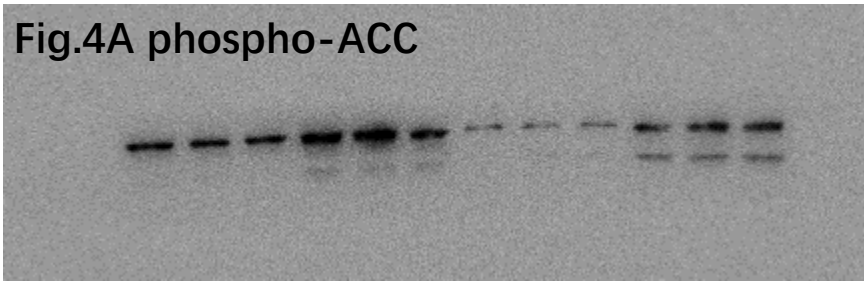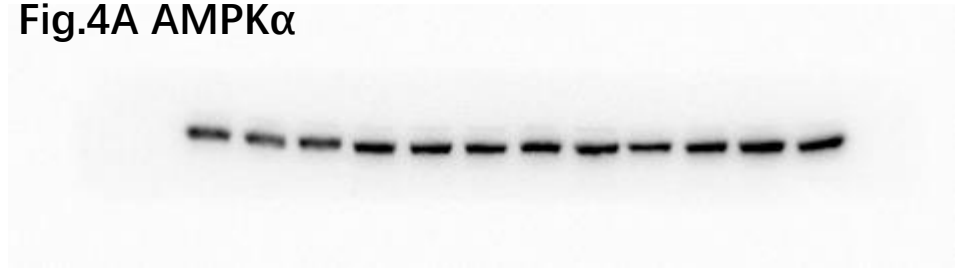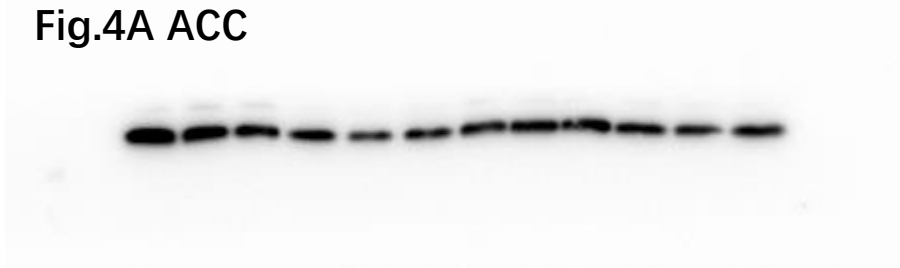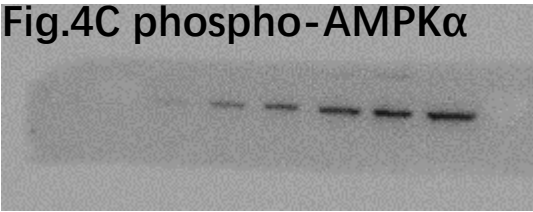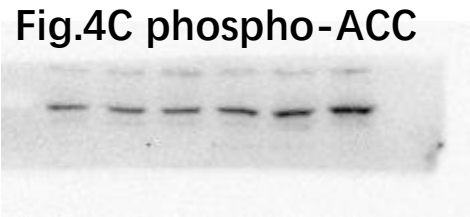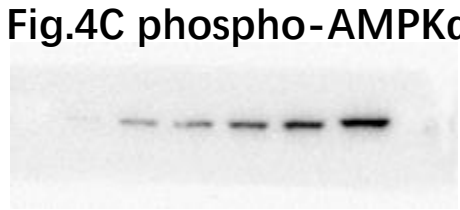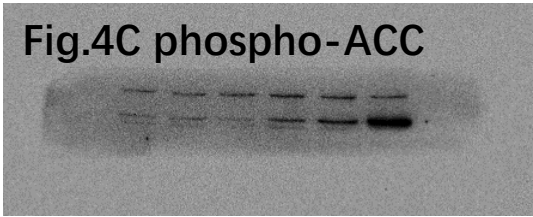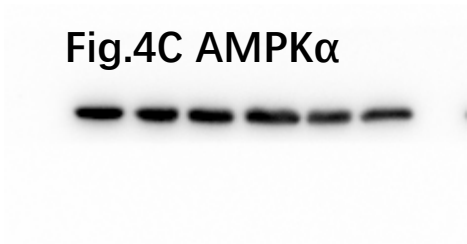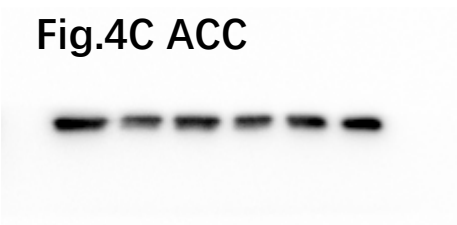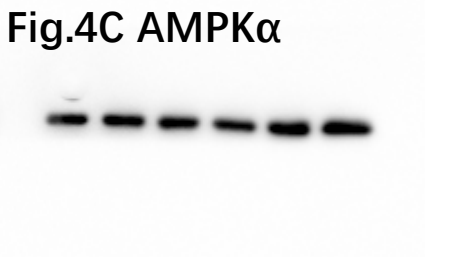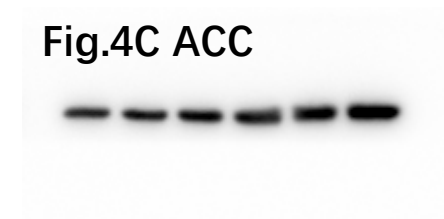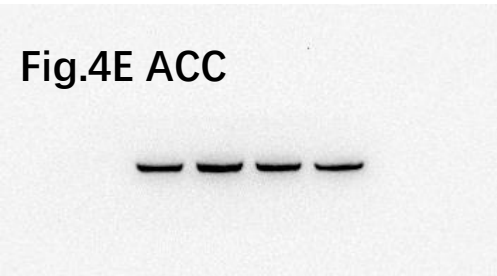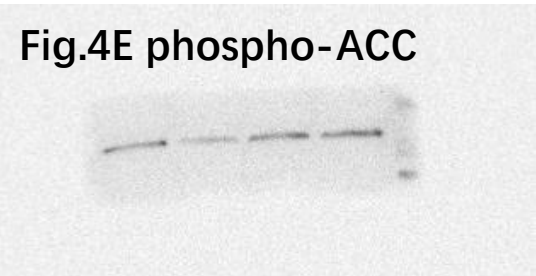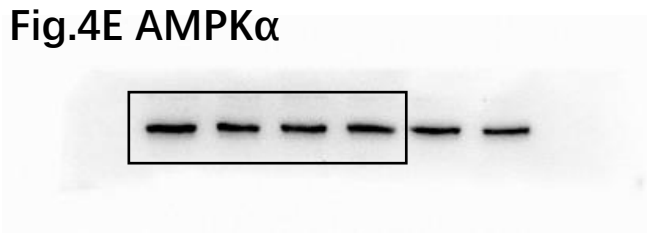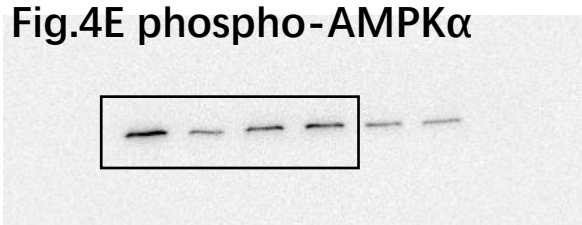

Supplement: Supplementary file 3 [file DataSheet1.PDF]
